# An evaluation of pre-registration research teaching provision for operating department practice students

**DOI:** 10.1177/17504589251326820

**Published:** 2025-03-28

**Authors:** Victoria Cadman, Adele Nightingale

**Affiliations:** 1 Sheffield Hallam University, Sheffield, UK; 2 University of Bolton, Bolton, UK

**Keywords:** Research, teaching, education, operating department practice, operating department practitioners

## Abstract

**Background:**

Understanding and engaging in and with research is a professional obligation of both student and registered operating department practitioners. This study was designed to explore how research is embedded into operating department practice undergraduate pre-registration curriculum.

**Method:**

Data collection took place via a questionnaire sent to programme leaders at Higher Education Institutions who deliver Health and Care Professions Council–validated operating department practice programmes. Overall, 30 higher education institutions were invited to participate, with 14 higher education institutions completing the questionnaire. The data were thematically analysed.

**Findings:**

It was identified that some aspects of research are embedded throughout operating department practice programmes, with a broad focus on research methodologies, methods and ethical considerations. The evidence supports the hypothesis that operating department practitioner students are undertaking primary and secondary research. However, there is limited evidence of dissemination. This work has identified the perceived barriers and challenges to embedding research in undergraduate programmes and the limited use of enrichment activities. This has led the authors to identify some areas worthy of further exploration and recommendations for the future.

## Introduction

The benefits of research and having research active staff who disseminate and embed research into their practice are well documented and related to staff morale and positive patient experience and outcomes (Boaz et al 2015, Council for Allied Health Professions Research (CAHPR) 2019). Therefore, it is imperative that the practice of operating department practitioners (ODPs) is informed by a robust evidence base and that student ODPs develop research skills, awareness and understanding in their pre-registration undergraduate (UG) studies (College of Operating Department Practitioners (CODP) 2018).

ODP programmes are all validated by the Health and Care Professions Council (HCPC) against the Standards for Education and Training (SETs) (Health and Care Professions Council (HCPC) 2018) and as such will attain a certain standard to support delivery. It is advocated as part of the SETs that all proposed programmes meet the CODP national curriculum (CODP 2018). Many, but not all higher education institutions (HEIs) programmes are endorsed by the CODP and while this may be considered good practice, it is not a prerequisite for delivery. As there is a national curriculum, there will be considerable similarities in the content of programmes; however, how they are delivered may be unique to that HEI. Both documents governing ODP education contain elements specifically related to research knowledge and skills, demonstrating it is an essential component of ODP pre-registration education.

The Community (formerly Council) for Allied Health Professions Research (CAHPR) position statement published in 2019 recognises that Allied Health Professions (AHP) pre-registration programmes need to ensure that their current curricula ‘are enabling students to become research aware’ (CAHPR 2019; 2). There is also a request that professional bodies review their expectations of pre-registration programmes. The Multiprofessional Practice-Based Research Capabilities Framework provides eight capability domains that can be used to support delivery towards meeting entry-level capabilities (NHS England 2024). It demonstrates how the domains develop as careers progress which is useful for emphasising research value and importance to students. Therefore, this work to evaluate ODP pre-registration research teaching is considered necessary, especially at a time when the CODP Curriculum Document is in its fifth year, with a timely review imminent (CODP 2018).

As described in both the CODP Curriculum and the HCPC Standards of Proficiency, it is evident that there is a prerequisite to have research embedded within the ODP programme outcomes (CODP 2018, HCPC 2023). It is also clear that there is a need for programme leaders to engage with research or publish as their contribution to the profession (CODP 2018). However, the latest ODP Workforce Report identified research as one of the areas of practice in which ODPs are underrepresented and requires further exploration to understand how UG programmes and delivery teams are contributing to research (Health Education England (HEE) 2023).

The aim of this project is to explore how research is embedded into the syllabi of ODP UG pre-registration programmes.

## Method

Ethical approval for undertaking the study was provided by Sheffield Hallam University (ER66527588) and also registered at University of Bolton. The evaluation was designed in a way that required one response from HEIs that are currently delivering an HCPC-validated course programme for ODP. These could be direct entry programmes (application via University and Colleges Application System (UCAS)), degree apprenticeship programmes or both. Given the specific nature of the respondents required, the CODP kindly circulated an invitation to ODP programme/course leads to participate through completion on behalf of their institution. Further information regarding the study, its purpose and how information would be shared was provided. The invitation letter contained the link to the form for completion. Consent was obtained in relation to participation in the study and how information would subsequently be used in line with ethical approval. The rest of the form contained questions relating to the type of course, research-specific modules, how research teaching was delivered across the course, enrichment activities relating to research and opportunity to provide further comment regarding research teaching and assessment for informing development. Responses from open questions were reviewed independently by both researchers and analysed thematically within each question. Agreement was reached regarding findings and evaluation, and these are presented. Illustrative quotes are provided verbatim from written responses.

## Results

Notably, 15 responses were received but on examination, it was found that one HEI had submitted twice; therefore, there were 14 responding institutions. These institutions covered a large geographical area across England and Wales, encompassing some of the largest education providers for ODP. Most of the responding institutions (nine) provide both course routes, with four providing only the direct entry route, and one course only having degree apprenticeship provision. All institutions responded that they provided a specific research module as part of their course programme(s), this was typically at Level 5 (second year of study). Thematic synthesis of the findings from open questions is provided below.

### Teaching and assessment

It is evident that there is variation in terms of research delivery in the first year of study with not all HEIs having research modules at Level 4, and those that do, often combine research with the professional practice element of ODP. This first year of study appears to introduce the students to the concept of evidence-based practice, the language of research, and comparing and contrasting research papers with a view to being a critical user of research. In addition, some HEIs recognise the need to support students with their academic writing skills, which is a fundamental aspect of their degree.

It is apparent at Level 5 that there is a focus on understanding research methodologies, methods and ethical considerations of research, all of which are undoubtedly important skills to acquire. There is a positive approach to undertaking literature searches and developing an understanding of critiquing tools:

. . .undertake a structured literature search to find a research paper of their choice which they then critically review using a relevant critiquing framework. . . (HEI 1)

. . . where a critical appraisal of a published article is assessed using the CASP tool. Articles are provided by the ML with either a qualitative or quantitative article being selected by the student. (HEI 10)

. . . assignment describing their literature search strategies, justification for choice of paper, its appraisal and how the findings might be applied to practice. (HEI 13)

However, the knowledge and skills developed in Level-5 modules do not support students to actively engage in research, with most HEIs providing response that allude to the development of awareness of theory, rather than an of application of theory to practice and engaging with data collection.

The majority of Level-6 modules, of both direct entry and apprenticeship programmes, had research embedded. A number of programmes described a dissertation module, with a small number recounting how their students undertook ‘primary research’ in their final year. Many of the programmes portrayed the completion of secondary research projects, with a number completing systematic reviews and empirical research.

This can be empirical research for publication in a journal or a literature review on a topic of their own choosing linked to perioperative practice. Most students undertake a literature review. (HEI 4)

Although a number of programmes detailed other research-related activity in the third year, this focused on quality improvement (QI) projects, service evaluations, independent projects and extended projects:

A quality improvement project or service evaluation which captures a critical exploration of the literature around a proposed change in practice. (HEI 6)

### Enrichment

While most existing definitions of enrichment activity come from school and further education, it would be fair to say that this is transferable to the higher education context. Therefore, enrichment activities are those that are organised and facilitated by the educational provider to enhance and extend, but is not part of, the formal academic curricula (Esmond et al 2024, Renzulli et al 2021, SQW 2024).

There were a number of alternative responses which detailed how research active ODPs were invited to deliver guest lectures to role model positive research behaviours. In addition, some responses specified how ODP students were encouraged to participate in ODP research projects undertaken by HEI programme teams, and the creative way support was provided for dissertations. While it is encouraging to see that some HEI programmes are attempting to generate a positive research culture, these activities may be seen as primarily related to creating a positive teaching and learning environment, rather that directly targeting ‘enrichment’. Some HEIs reported encouraging students to access external opportunities but stated that uptake to such activity was low:

We have offered internal internships for undergraduates to become involved in research projects (but uptake was poor). (HEI 1)

### Barriers and facilitators

It is recognised by some universities that there are challenges for ODP students to undertake any research project that requires ethical approval, either through the Integrated Research Application System (IRAS) or HEI ethical approval process:

It is helpful for students to engage in research, but ethics is always seen as problematic. This is either because the NHS ethics is too long and onerous for undergraduate students to be able to complete, or the University ethics process struggles to approve a large number of undergraduate ethics applications in sufficient time for the students. (HEI 1)

Meandering through the issues around university + NHS ethics approval. . . (HEI 7)

Other challenges detailed that from a student’s perspective, research is seen as undervalued in clinical practice:

Biggest barrier to learning appears to be negativity towards research that may be culturally driven (from workplace). (HEI 5)

It is challenging to engage learners with research topics and for them to see the value/relevance of the module for their practice. (HEI 7)

Profile of the need for research in our profession with role modelling and curriculum design. Too many opinions that we don’t need to do it or there is no need. (HEI 8)

In addition, the concepts covered within research are perceived as problematic for students to grasp resulting in anxiety:

From my own experience, I feel it is an abstract concept for many learners. . .I think that it also helps having ODP lecturers who are research active because this also makes the topic less abstract. (HEI 13)

Breaking down research into smaller, more attainable, simplified ‘chunks’ helps to relieve the anxiety that some students face with research. (HEI 3)

ODP research activity is an influencing factor in teaching delivery with reference to research active ODP staff using their experience as a positive role model, whereas other responses commented on a lack of ODP research knowledge base to draw on:

It would be helpful if students had a specific ODP knowledge base to reference but, I appreciate that this is unlikely to happen in the short term. (HEI 10)

One notable barrier identified from responses is a misunderstanding of the nuanced difference between research teaching and evidence-based practice teaching.

## Discussion

The CAHPR (2019) position statement articulates that there is a strong need for AHP activity to be evidenced-based and that students have the ability to critique research, enabling them to embed research in to their practice. The qualitative data acquired from this study informed the authors of the current state of research education for ODP students in 14 HEIs across the United Kingdom. The variability identified across the Level-4 modules does not appear to be unique to ODP. A recent study by Pagnamenta et al (2022) exploring research training for UG speech and language therapists (SLT) found similar findings. Pagnamenta et al (2022) found that in UG SLT students, there was variation in their confidence to engage with research, in addition to their lack of research awareness. This led to the conclusion that a lack of confidence and awareness has direct implications when embedding research into clinical practice (Pagnamenta et al 2022)

There is an expectation from CAHPR (2019: 3) that UG pre-registration students should have the ability to participate in ‘methodologically robust research’. At Level 5, there is a positive approach to undertaking literature searches and developing an understanding of critiquing tools. This focused largely on developing knowledge in preparation for Level 6 where some form of extended project/dissertation is completed. While this is encouraging to see and is an exceptionally favourable approach to embedding research into pre-registration UG programmes, it does generate further related concerns. If we have a large number of ODP students across multiple universities undertaking primary and secondary empirical research and systematic reviews, where is this new knowledge and contribution to perioperative research being published and further disseminated? There is clearly a significant amount of research and QI work occurring in HEIs in the UK-related to perioperative practice; however, to the best of the authors’ knowledge, evidence on dissemination of this work and of its impact on practice is lacking. It could be argued that research that is not disseminated and shared defeats the object of research; however, it is recognised that there are ethical considerations related to dissemination and implementation of research findings (Tetteh et al 2023). This is a subject worthy of further exploration and may be recognised as a barrier to dissemination. While the authors truly advocate for research at all levels of ODP, we would suggest that HEIs need to support their students to disseminate their research in an ethical way, including reporting results accurately, timely and transparently while declaring conflicts of interest (Derman & Jaeger 2018, DuBois & Prusaczyk 2017). Research modules should be designed to facilitate this through the use of credible projects rather than ‘recycling’ set projects that do not allow students to develop skills in identifying gaps and limit innovation. This could be a positive opportunity for HEI staff to jointly publish with students which would create a repository of ODP research, developing the capacity and capability of ODPs in research and also contribute to the Research Excellence Framework (REF). This is a concept supported by Adebisi (2022) who suggests that UG research initiatives have the power to positively benefit programmes, faculties and institutions. Furthermore, Jansen et al (2015) suggests that in UG nursing programmes, there is increased engagement in faculty research projects, highlighting the need to immerse UG students in all aspects of the research process. This approach is claimed to offer richer learning experiences and creates students who are enthusiastic in their approach to research and contributing to the knowledge development in their profession (Jansen et al 2015)

Level-6 study is designed to create a systematic understanding of the perioperative environment creating practitioners who can critically analyse and evaluate extensive sources of data from a wide range of perspectives leading to detailed and sustained arguments. Practitioners at this level will have developed a critical lens through which to view their own work and that published by others. This is one of the key findings of the Council of Deans for Health paper ‘Becoming Research Confident’ published in 2019. It is suggested that ‘all health care professionals should be able to critically assess and use evidence that underpins practice’ (McCormack et al 2024: 4). ODP students at the forefront of our profession should be encouraged and supported to clearly articulate dissemination strategies for their work. This would not only have the potential to enhance patient care and experience but also to encourage them to celebrate their success and be proud of their contribution to research and innovation. This foundation will be the basis on which the ODP profession will continue to evolve and establish themselves as a profession that contributes to quality research.

While students undertaking research is absolutely seen as a challenge, some universities are embracing that challenge and overcoming it. Therefore, it is imperative that HEIs start to share how they have overcome these challenges. All universities work within the Framework for Research Ethics (UK Research and Innovation (UKRI) 2024), a framework that ensures all research follows the same guiding ethical principles. Therefore, if one university has a process that supports students’ ethical approval to be undertaken in a timely manner, then other universities should theoretically be able to commit to the same standards and timelines. There is also potential opportunity here for universities to collaborate and provide larger-scale staff-led projects that students can be involved in for their dissertations/extended projects.

McCormack et al (2024) found that not all staff recognise the importance of research and this is a barrier to integrating research in to UG programmes. They suggest there are negative perceptions of research in practice which were reported as problematic; however, it is within our gift to create students and newly qualified ODPs to challenge this dated mantra. Recently a study by Conway et al (2024) suggested that theatre practitioners who have studied at Masters level and have engaged post-registration with research have a more positive attitude [to research]. There is so much activity taking place with regards to the AHP research, that now is an exciting time to change the narrative and support of the ODP profession to be part of the AHP research community. The AHP Research and Innovation Strategy (HEE 2022) offers a strong foundation on which to now build, challenge and embed AHP research. The CODP, is an active member of the CAHPR, demonstrating that professionally ODP is working towards a vision shared by AHPs in England. It is anticipated that this will have a positive effect on the profession of ODP as it will afford opportunities for multi-profession research. A recent Delphi study undertaken by Nightingale et al (2025) established research priorities for the ODP profession. This gives the profession direction, a sense of purpose but an equally important opportunity to work with ODP students, other professions, HEIs and NHS trust partners to encourage a collaborative approach to research and build on this aspect of the Four Pillars of Practice (HEE 2017).

Enrichment activities are those activities which occur outside of timetabled activity and benefit the student both personally and academically. At UG level, it is expected that students are motivated to engage with enrichment activities. While the ODP programme is highly specialised and focused on the perioperative environment, it is anticipated that students would be motivated and inspired to delve deeper in to the many facets of perioperative medicine, care and environment, and wider, contemporary healthcare issues thus exploring new areas of knowledge beyond the curriculum. Enrichment affords the benefits of engaging with challenging material and stimulating curiosity outside of the timetable. However, the data captured through this study demonstrate that there may be a lack of enrichment activity offered to ODP students, with only one HEI articulating that they offer a ‘Journal Club’ and ‘Research Workshops’. Mass-Hernández et al (2022) advocates the use of research interest groups facilitated by lecturers and research active staff to support students as mentors. They suggest the use of research activities, including seminars and workshops, enhance student’s opportunities to contribute to research. Enrichment activities take commitment and enthusiasm to engage with from both a student’s perspective and also from a lecturer’s perspective with one HEI, when asked about enrichment activity stating ‘they do not do anything above and beyond the curriculum’. From a student’s perspective, if no extra-curricular activity is offered, there is reduced opportunity for them to engage wider, thus creating a curriculum-led programme rather than one which provides the added value of enrichment. It has been identified in UG medical education that those students who participate in research projects as UG are more likely to publish higher numbers of, and better-quality papers throughout their careers suggesting that active engagement in research encourages them to embed research and subsequent publications into their post-graduate practice (Mass-Hernández et al 2022). However, this concept is challenged by Riiser et al (2023) who conclude from their recent scoping review that more research is needed across healthcare programmes to understand if those students who do actively engage as UG subsequently participate in further research in the healthcare careers. This creates discourse across professions and one which is worthy of further research.

From the data collected, some misunderstanding was noticed in relation to research that potentially restricts growth in the research capability of the profession. ‘Evidence-based practice’ and ‘research’ were used interchangeably and this impacts on understanding as evidence-based practice is not itself research. Differentiating between these terms, in addition to QI is important when we consider the contribution to new knowledge, innovation and improvements. As addressed earlier, there is an expectation that ODPs will contribute to these processes; therefore, the nuances of each needs to be understood (Conner 2014).

While the data collected has been useful to evaluate the current picture in relation to pre-registration teaching provision, there are some limitations to this work. There are 30 providers of pre-registration training in the United Kingdom, yet only 14 HEIs responded, which was disappointing for the authors. The authors recognise that a higher response rate would have enabled them to generate a more accurate picture to inform curriculum development.

## Recommendations

There is potential to enhance the experience of both staff and students in terms of research teaching delivery with several aspects that require additional research to be undertaken to inform work and fully enable support for staff at all levels of practice in the development of ODP research capability. Suggested areas for research include the following:

- Exploring the perceptions of research held by students, HEI staff and staff in clinical practice into the value of research.
- Barriers and facilitators to research teaching.
- Perceptions of research career development.

We also recommend that HEIs consider ways in which to offer true enrichment activity relating to research.

## Conclusion

Findings from this evaluation study indicate that on the whole, HEIs provide comparative research teaching to meet standards. There is little evidence of enhanced provision or enrichment opportunities which would help facilitate a more positive view of research and alter perceptions to build capacity. There is evidence which suggests those professions and NHS trusts who actively promote research by their staff, create better outcomes for their patients. Therefore, it is imperative that we build a profession that has research as the golden thread at individual, team and organisational level to ensure we influence patient safety and progression in practice.

Strengthening the development of research capability at UG level has the potential to support the transition of ODPs from users of research to being recognised as research-active.

## References

[bibr1-17504589251326820] Adebisi AY 2022 Undergraduate students' involvement in research: Values, benefits, barriers and recommendations Annals of Medicine and Surgery 81 104384 36042923 10.1016/j.amsu.2022.104384 PMC9420469

[bibr2-17504589251326820] Boaz A Hanney S Jones T , et al 2015 Does the engagement of clinicians and organisations in research improve healthcare performance: A three-stage review BMJ Open 5 e009415 10.1136/bmjopen-2015-009415 PMC4680006 26656023

[bibr3-17504589251326820] College of Operating Department Practitioners (CODP) 2018 Bachelor of science in operating department practice curriculum document [Online] Available from: https://www.unison.org.uk/content/uploads/2018/09/CODP-BScHons-in-ODP-Curriculum-Document-Sept-2018.pdf [Accessed July 2024]

[bibr4-17504589251326820] Council for Allied Health Professions Reserach (CAHPR) 2019 Council of Allied Health Professions Position statement: Developing research skillswithin AHP pre registration education. Available from: cahpr_position_statement_research_skills_final.pdf [Accessed March 2025]

[bibr5-17504589251326820] Conner B 2014 Differentiating between research, evidence-based practice and quality improvement. Differentiating research, evidence-based practice, and quality improvement [Online] Available from: https://www.myamericannurse.com/differentiating-research-evidence-based-practice-and-quality-improvement/ [Accessed October 2024]

[bibr6-17504589251326820] Conway N Bradburn A Howcutt S 2024 Exploration of attitudes towards research: Operating department practitioners and theatre nurses Journal of Perioperative Practice Epub ahead of print 19 December DOI: 10.1177/17504589241301204 PMC12712228 39698898

[bibr7-17504589251326820] Derman RJ Jaeger FJ 2018 Overcoming challenges to dissemination and implementation of research findings in under-resourced countries Reproductive Health 15 (Suppl. 1) 86. 29945654 10.1186/s12978-018-0538-z PMC6019998

[bibr8-17504589251326820] DuBois JM Prusaczyk B 2017 Ethical issues in dissemination and implementation research. In: Brownson RC Colditz GA Proctor EK (eds) Dissemination and Implementation Research in Health: Translating Science to Practice 2nd ed New York, Oxford AcademicAcademic [Online] Available from: 10.1093/oso/9780190683214.003.0004 [Accessed January 2025]

[bibr9-17504589251326820] Esmond B Kaur B Wood M Blake H Atkins L 2024 Valuing enrichment: Final report [Online] Available from https://d4hfzltwt4wv7.cloudfront.net/uploads/files/Valuing-Enrichment-Report-30.04.24.pdf [Accessed February 2025]

[bibr10-17504589251326820] Health and Care Professions Council (HCPC) 2018 Standards for Education and Training [Online] Available from: https://www.hcpc-uk.org/globalassets/resources/guidance/standards-of-education-and-training-guidance.pdf [Accessed October 2024]

[bibr11-17504589251326820] Health and Care Professions Council (HCPC) 2023 Standards of proficiency: Operating department practitioners [Online] Available from: https://www.hcpc-uk.org/standards/standards-of-proficiency/operating-department-practitioners/ [Accessed August 2024]

[bibr12-17504589251326820] Health Education England (HEE) 2017 Multi-professional framework for advanced clinical practice in England [Online] Available from: https://www.hee.nhs.uk/sites/default/files/documents/multi-professionalframeworkforadvancedclinicalpracticeinengland.pdf [Accessed September 2024]

[bibr13-17504589251326820] Health Education England (HEE) 2022 Allied Health Professions’ Research & Innovation Strategy for England [Online] Available from: https://www.hee.nhs.uk/sites/default/files/documents/HEE%20Allied%20Health%20Professions%20Research%20and%20Innovation%20Strategy%20FINAL_0.pdf [Accessed July 2024]

[bibr14-17504589251326820] Health Education England (HEE) 2023 National Operating Department Practitioner (ODP) workforce programme 2021-22: Scoping the future of the ODP workforce, Full report [Online] Available from: https://www.hee.nhs.uk/sites/default/files/documents/ODP%20full%20report.pdf

[bibr15-17504589251326820] Jansen DA Jadack RA Ayoola AB , et al 2015 Embedding research in undergraduate learning opportunities Western Journal of Nursing Research 37 (10) 1340–1358 25694176 10.1177/0193945915571136

[bibr16-17504589251326820] Mass-Hernández LM Acevedo-Aguilar LM Lozada-Martínez ID , et al 2022 Undergraduate research in medicine: A summary of the evidence on problems, solutions and outcomes Annals of Medicine & Surgery 74 103280 35127067 10.1016/j.amsu.2022.103280 PMC8807964

[bibr17-17504589251326820] McCormack B Baltruks D Cooke R 2024 Becoming research confident: Research in pre-registration curricula for nursing, midwifery and allied health programmes [Online] Available from: https://councilofdeans.org.uk/wp-content/uploads/2019/05/CODH.RIPR_.report_v3-002.pdf [Accessed October 2024]

[bibr18-17504589251326820] NHS England 2024 The centre for advancing practice: Multiprofessional practice-based research capabilities framework [Online] Available from: https://advanced-practice.hee.nhs.uk/our-work/research/multi-professional-practice-based-research-capabilities-framework/ [Accessed July 2024]

[bibr19-17504589251326820] Nightingale A Cadman V McIntryre V , et al 2025 Operating department practitioner’s research priorities: A Delphi study 10.1177/17504589251330423 PMC12913671 40260607

[bibr20-17504589251326820] Pagnamenta E Longhurst L Breaks A , et al 2022. Research priorities to improve the health of children and adults with dysphagia: A national institute of health research and royal college of speech and language therapists research priority setting partnership. BMJ Open 12(1) 10.1136/bmjopen-2021-049459 PMC8796217 35078835

[bibr21-17504589251326820] Renzulli J Reis S Brigandi C 2021 Enrichment theory, research, and practice In Critical Issues and Practices in Gifted Education London, Routledge pp 185–199 [Online] Available from https://www.researchgate.net/publication/354502967_Enrichment_Theory_Research_and_Practice [Accessed January 2025]

[bibr22-17504589251326820] Riiser K Kalleson R Holmen H Torbjornsen A 2023 Integrating research in health professions education: A scoping review BMC Medical Education 23 653 37684582 10.1186/s12909-023-04615-4 PMC10492286

[bibr23-17504589251326820] SQW 2024 Youth enrichment: Discovery phase: Discussion paper submitted to the Department for Culture, Media and Sport by SQW [Online] Available from: https://assets.publishing.service.gov.uk/media/6723985dbce643d1194f99e1/Youth_Enrichment_Report_-_DCMS_Final-accessible__1_.pdf [Accessed February 2025]

[bibr24-17504589251326820] Tetteh EK Geng EH Huffman MD 2023 Developing ethical standards for dissemination and implementation research: A roadmap for consensus and guidance Implementation Science Communications 4 132 37932842 10.1186/s43058-023-00514-3 PMC10629054

[bibr25-17504589251326820] UK Research and Innovation (UKRI) 2024 Framework for research and Ethics – UKRI [Online] Available from: https://www.ukri.org/councils/esrc/guidance-for-applicants/research-ethics-guidance/framework-for-research-ethics/ [Accessed October 2024]

